# Antiadherent AgBDC Metal–Organic Framework Coating for *Escherichia coli* Biofilm Inhibition

**DOI:** 10.3390/pharmaceutics15010301

**Published:** 2023-01-16

**Authors:** Ana Arenas-Vivo, Vanessa Celis Arias, Georgiana Amariei, Roberto Rosal, Isabel Izquierdo-Barba, Tania Hidalgo, María Vallet-Regí, Hiram I. Beltrán, Sandra Loera-Serna, Patricia Horcajada

**Affiliations:** 1 Advanced Porous Materials Unit (APMU), IMDEA Energy Institute, Avda. Ramón de la Sagra 3, 28935 Móstoles, Spain; 2 Basic Science Department, Metropolitan-Azcapotzalco Autonomous University (UAM), Av. San Pablo No 180, Col. Reynosa-Tamaulipas, Ciudad de México 02200, Mexico; 3 Department of Chemical Engineering, University of Alcalá, 28871 Alcalá de Henares, Spain; 4 Pharmaceutical Chemistry Department, Faculty of Pharmacy, University Complutense of Madrid, Hospital 12 de Octubre i+12 Institute for Health Research, 28040 Madrid, Spain; 5 CIBER of Bioengineering, Biomaterials and Nanomedicine (CIBER-BBN), 28040 Madrid, Spain

**Keywords:** Metal–organic frameworks, silver, antifouling, bactericide, biofilm, *Escherichia coli*

## Abstract

Surface microbial colonization and its potential biofilm formation are currently a major unsolved problem, causing almost 75% of human infectious diseases. Pathogenic biofilms are capable of surviving high antibiotic doses, resulting in inefficient treatments and, subsequently, raised infection prevalence rates. Antibacterial coatings have become a promising strategy against the biofilm formation in biomedical devices due to their biocidal activity without compromising the bulk material. Here, we propose for the first time a silver-based metal–organic framework (MOF; here denoted *AgBDC*) showing original antifouling properties able to suppress not only the initial bacterial adhesion, but also the potential surface contamination. Firstly, the AgBDC stability (colloidal, structural and chemical) was confirmed under bacteria culture conditions by using agar diffusion and colony counting assays, evidencing its biocide effect against the challenging *E. coli*, one of the main representative indicators of Gram-negative resistance bacteria. Then, this material was shaped as homogeneous spin-coated AgBDC thin film, investigating its antifouling and biocide features using a combination of complementary procedures such as colony counting, optical density or confocal scanning microscopy, which allowed to visualize for the first time the biofilm impact generated by MOFs via a specific fluorochrome, calcofluor.

## 1. Introduction

Today more than ever, the threat of bacterial infections is still an unresolved global issue, perpetrating more than 75% of human infectious diseases (e.g., tuberculosis, tetanus, cholera, etc.) [1]. They are mainly associated with the microbial dissemination on different surfaces, leading to large contamination due to the potential biofilm formation in that action place, which is still a current pressing concern [2]. Biofilms are complex microenvironments composed of colonies of microorganisms protected by extracellular polymeric substances (EPS), in which the bacteria are able to communicate and interact between them, generating strategies to defend themselves against any type of external stresses [3]. This harsh microbial environment is one of the reasons behind the complexity of inhibiting biofilms as well as the rising tendency to generate antimicrobial resistances against traditional chemical antibiotics [4]. Thus, shedding light on the biofilm emergence could enhance efficient treatments.

Biofilm formation entails four different stages: (i) initial adhesion by planktonic bacteria (in suspension) approaching the substrate; (ii) colony-forming units (CFUs) with a proliferation step; (iii) a subsequently maturation of the biofilm, associated with an EPS generation; and finally, (iv) the biofilm redispersion, completing the entire cycle. Many of the proposed therapeutic strategies have targeted each of these formation stages for their complete eradication [5]; however, just the ones tackling the first cycle phases seem to be more effective (e.g., limiting bacterial adhesion, still in a reversible stage, or preventing the EPS generation) since treating already-contaminated surfaces could lead to an incomplete bacterial death, releasing a larger amount of planktonic bacteria to this microenvironment [2].

Among other recent alternatives, the use of antibacterial coatings has become an upward trend in the scientific community since thin films can provide the desired biocidal effect to the surface without compromising the bulk material features. The design of these linings is based on three main approaches, such as the active ingredients (AIs) release, contact-killing or antiadhesion/bacteria repelling [6]. Despite the reported progress on the topic, there are still some drawbacks to be overcome (e.g., limited reservoirs, lack of long-term effects, potential cytotoxicity, associated inflammatory responses), which has led to novel efficient approaches. Herein, we propose the use of metal–organic frameworks (MOFs) as surface antimicrobial coatings with potential antiadherent properties. MOFs are a promising family of porous crystalline hybrid solids, comprising inorganic nodes (e.g., atoms, clusters, chains) and organic polycomplexant linkers (e.g., carboxylates, phosphonates, azolates) that assemble into multidimensional periodic lattices [7]. Additionally, MOFs have been studied as antibacterial platforms due to their inherent properties (e.g., structural and compositional tunability, ordered distribution of active sites, exceptional porosity) [8,9,10]. Compared with other classic porous materials, these outstanding features result from: (i) their chemical/structural versatility, which permits a suitable biocompatibility upon chemical design; (ii) an ideal hydrophobic/hydrophilic internal microenvironment conveniently adapted to host a very broad variety of active ingredients (e.g., biological gases, cosmetics, enzymes, nucleic acids, drugs); (iii) releasing them in a controlled manner under physiological conditions; (iv) easy and scalable synthesis, following green methods with high yields; (iv) *in vivo* safety; and (v) their external surface engineering, endowing further multifunctional abilities, such as furtivity, targeting and imaging, or enhancing their stability (chemical/structural/colloidal) under biorelevant media [11]. In addition, taking into account the well-known biocide character of silver (used in commercial solutions) [11,12,13], it is no wonder that many publications proposed the use of Ag-based MOFs for antibacterial therapy (see a brief summary in Supplementary Materials, Table S2 [14,15,16,17,18,19,20,21,22,23,24,25,26,27,28,29,30,31,32,33,34,35]). Focusing just on the state of the art of those works using Ag-MOF coatings against bacterial biofilm (e.g., AgBTC [14,15], AgBDC-NH_2_ [16,17], Ag-imidazolate [18,33], Ag_2_[HBTC][im] [19]), a great inhibition of the colony-growth viability (>80%) was achieved. However, the direct observation of these reported surfaces by means of confocal microscopy showing an active biofilm disruption has not been performed. Therefore, besides their demonstrated biocide properties, no active antifouling Ag-based MOF layers have been yet accomplished.

To the best of our knowledge, there are very few reports targeting the study of MOF coatings for the inhibition of the initial bacterial adhesion, further preventing the biofilm development. Just Zang [36] and Gu [37] considered in their investigations the quantification of the potential adhesion reduction in presence of the MOF coating by determining the CFU via plate counting of the sessile bacteria in the biofilm (anchored to a substrate). In spite of this, their work lacks experimental surface visualization or even the verification of the adhesion process, hindering the support of the bacteria viability. Other work by Lui et al. [38] also attempted to lessen the bacteria attachment by using a composite of iron terephthalate MIL-88B with Ce. This composite was applied for biofilm dispersion and treatment of previously contaminated surfaces, not as biofilm prevention implementation. Finally, another strategy found in the literature, followed by one of the authors of the present work, involved the irradiation of an MOF coating to activate the dispersion of the attached bacteria [39,40]. Although the photoactive-MOF layer reduced the initial number of colonies adhered to the substrate, complete antifouling effect was not achieved until irradiation.

In this case, for the first time, the authors aimed to develop here an active MOF thin film with full antifouling properties that will suppress initial bacterial adhesion, hampering the biofilm formation and, consequently, the surface contamination. The selected MOF structure, denoted AgBDC, was previously reported by Sun et al. [41], being optimized here with a new-fashioned protocol with a simpler and faster reaction for the upscale attainment of AgBDC submicrometric particles (~250 nm). The colloidal, structural and chemical stability of AgBDC was verified under bacteria culture conditions. In a bacteria biofilm model, *Escherichia coli* (*E. coli*) biofilms were chosen since they are the major cause of morbidity and mortality behind intestinal infections, are the reason behind the spread of nosocomial infections derived by the contamination of diverse healthcare equipment (e.g., medical devices, joints, shuts, prosthetic grafts) and are one of the most infectious bacteria along with *Staphylococcus aureus* (*S. aureus*) and *Pseudomonas aeruginosa* [42,43]. In addition, *E. coli* is a Gram (−) facultative anaerobic bacterium, which makes it one of the most adaptable organism that could be grown in laboratory conditions, making it an ideal candidate to test the biofilm treatment [44]. Practically, the biocide activity of AgBDC against planktonic *E. coli* was identified through the agar diffusion assays along with its minimum inhibitory concentration (MIC, ~100 ppm) determined by colony counting. Then, homogeneous spin-coated AgBDC thin films were prepared to investigate their antifouling and biocide properties by a combination of different complementary techniques (confocal microscopy, scanning electron microscopy (SEM), colony counting, optical density (OD_600_)). Finally, it should be noted that this this study used, for the first time, a specific fluorochrome (calcofluor) for the direct visualization of the EPS presence via confocal laser scanning microscopy, unveiling and further confirming the absence of biofilm formation.

## 2. Materials and Methods

### 2.1. Materials

All the chemicals were used as received. Benzene 1,4-dicarboxylic acid (BDC, MW: 166.13 g·mol^−1^, 98% grade), sodium hydroxide (NaOH, MW: 40 g·mol^−1^, 98% grade) and silver nitrate (AgNO_3_, MW: 169.87 g·mol^−1^, 99% grade) were purchased from Sigma-Aldrich (St. Louis, MO, USA). Anhydrous ethanol (EtOH, 99% of purity, Aldrich) and deionized (DI) water were used as solvents. For the preparation of the bacteriological broth culture known as nutrient broth (NB), beef extract and peptone (pH 7–7.2) were purchased from Condalab (Torrejón de Ardoz, Madrid, Spain).

### 2.2. AgBDC MOF Synthesis and Characterization

#### 2.2.1. Synthesis of MOF

AgBDC synthesis ([AgC_4_H_2_O_2_], MW: 190 g·mol^−1^) was optimized from a previous protocol [41]. Briefly, BDC (0.5 g, 3 mmol) and NaOH (0.3 g, 7.5 mmol) were dissolved in 80 mL of deionized (DI) water in a round bottom flask. Then, a 20 mL EtOH solution of AgNO_3_ (0.51 g, 3 mmol) was added dropwise over the previous solution, stirring at room temperature (RT) during 24 h. The product was recovered by centrifugation (4032× *g* RCF, relative centrifugal force, 10 min) and dried out at 50 °C during 12 h. Yield: 43%, dry basis corrected through thermogravimetric analysis (TGA).

For the formation of AgBDC thin-film coatings, a 10 mL water suspension with 25 mg of AgBDC was prepared. With the assistance of a spin coater (SPIN150i, POLOS, program used: 510 rpm, period: 200 ms, amplitude: 90°, rotation time: 0.0/30.0 ss.s), 100 µL of the abovementioned suspension were deposited over one side of 13 mm diameter cover glass discs (VWR, Darmstadt, Germany) and were dried at RT for analyzing antifouling bactericidal activity (concentration = 1.9 µg·mm^−2^, or 0.25 mg per disc) (see Figure S5).

#### 2.2.2. MOF Characterization

X-ray powder diffraction (XRPD) patterns of AgBDC were collected in an Empyrean PANalytical^®^ powder diffractometer (PANalytical, Lelyweg, The Netherlands) in reflexion mode with a Cu Kα = 1.5406 Å. The XR diagrams were carried out with a 2ϴ scan between 3–90°, with a step size of 0.013° and a scanning speed of 0.1°·s^−1^. The Fourier transform infrared (FTIR) spectra (4000–650 cm^−1^) were obtained with a resolution of 2 cm^−1^ at RT on a Bruker Tensor-27 spectrometer (Bruker, Billerica, MA, USA), fitted with a DTGS detector (Bruker, Billerica, MA, USA). The FTIR spectra were recorded through the attenuated total reflectance (ATR) technique. Thermogravimetric analyses (TGA) were carried out in an SDT Q-600 thermobalance (TA Instruments, New Castle, DE, USA) with a general heating profile from 30 to 600 °C with a heating rate of 5 °C·min^−1^ under air using a flux of 100 mL·min^−1^. Note here that an air atmosphere was used to ensure the ligand decomposition and departure, as frequently used for MOF materials [45,46]. Quantitative determination of Ag content of the samples was conducted with inductively couple plasma optical emission spectroscopy (ICP-OES) on an Optima 3300 DV (PerkinElmer, Waltham, MA, USA); samples were thermally treated at 80 °C before digestion with HF and HNO_3_. Scanning electron microscopy (SEM) images of the AgBDC solid were taken with a Supra 55 VP (Carl Zeiss, Jena, Germany) field emission gun (FEG)-SEM after metallization of the samples. Dynamic light scattering (DLS) and ξ-potential (ZP) was registered via electrophoretic light scattering (ELS) using a Zetasizer Nano series Nano-ZS (Malvern Instruments, Worcestershire, UK). The particles were dispersed in the liquid media (methanol, ethanol, acetonitrile and water) at a concentration of 0.1 mg·mL^−1^ using an ultrasound tip (UP400S, Hilscher, Teltow, Germany) with 30% amplitude for 40 s.

### 2.3. MOF Stability Performance

#### 2.3.1. AgBDC MOF Suspension

Taking into account the AgBDC bacteriological application, both structural and colloidal stability of MOF suspensions in DI H_2_O and NB were assessed. For XRD analysis, AgBDC suspensions (4000 ppm) were incubated for 18 h at 37 °C. The solid was recovered by centrifugation (4032× *g* RCF, 10 min) and it was dried at 50 °C prior to the analysis. Particle size and ZP of AgBDC suspensions were evaluated by DLS measurements during different time points (0, 0.25, 0.5, 1, 2, 5, 8 and 18 h) under stirring conditions (2000 ppm).

#### 2.3.2. AgBDC MOF Thin Film

The adhesion stability of AgBDC thin film to the glass substrate was monitored over the time (up to 7 days) in 2.5 mL of NB at 37 °C (at 100 ppm of AgBDC), determining particle size and ZP by DLS in contact with this NB medium. Additionally, MOF chemical stability was analyzed by means of the BDC ligand release (wt.%) to the NB media, quantified via high-performance liquid chromatography (HPLC): a reversed phase HPLC Jasco LC-4000 series system, equipped with a PDA detector MD-4015 and a multisampler AS-4150 controlled by ChromNav software (Jasco Inc., Tokyo, Japan). A purple ODS reverse-phase column (5 µm, 4.6 × 150 mm^2^, Análisis Vínicos, Tomelloso, Spain) was employed, using an isocratic condition, with the flow rate at 1 mL·min^−1^, injection volume of 30 µL and the column temperature fixed at 25 °C. The mobile phase was based on a mixture of 50:50 MeOH:phosphate-buffered solution (PBS; 0.04 M, pH = 2.5) with a retention time (Rt) and maximum of absorption at 3.10 min and 240 nm, respectively (see Supplementary Materials, Figure S7). The PBS preparation (0.04 M, pH = *2.5*) was based on 0.02 mol (2.4 g) of NaH_2_PO_4_ and 0.02 mol (2.84 g) of Na_2_HPO_4_ were dissolved in 1 L of DI water. The pH was then adjusted to 2.5 with H_3_PO_4_ (≥85%). Given the low solubility of the BDC linker in aqueous solution and in order to avoid any potential underestimation during the stability test, 1.5 mL of the contact medium were taken after diverse time points (0.75, 2, 4 and 7 days), being evaporated at 100 °C. Afterwards, they were solubilized in 1.5 mL of methanol (MeOH), making them ready for HPLC analysis.

### 2.4. Bactericidal Activity and Biofilm Inhibition

#### 2.4.1. Microorganism Tests

*Escherichia coli* (*E. coli*) strain (CET 516, strain designation ATCC 8739) was employed to develop microbiological tests as a representative of biofilm-forming bacteria. They were kept frozen (−20 °C) until use. The microorganism was activated by growing it in 25 mL Erlenmeyer of nutrient broth (NB, no Cl, beef extract 5 g·L^−1^, peptone 10 g·L^−1^, pH = 7–7.2) under stirring conditions (100 rpm) at 37 °C. Inoculums were diluted with fresh NB_l_ at 10^6^ bacteria·mL^−1^ (tracked by measuring optical density at 600 nm, OD_600_, with a Shimadzu UV-1800 spectrophotometer (Shimadzu, Kyoto, Japan)) to preserve the exponentially growing phase of the microorganisms during the total incubation time. For comparison purposes, *Staphylococcus aureus* (*S. aureus*) (CETC 240, strain designation ATCC 6538P) was also cultivated in the same manner.

#### 2.4.2. Inhibition Halo Experiments

An antimicrobial effect was firstly evaluated using the agar diffusion method. For the diffusion tests, 20 ppm of MOF AgBDC were placed on the surface of soft agar plates (Petri dishes, Ø = 9 cm), previously inoculated with 0.6 mL of 10^6^ colony-forming units (CFU) mL^−1^ of *E. coli* and *S. aureus* bacterial suspensions, respectively, for 20 min at RT. They were incubated for 7 days at 37 °C, and the plates were digitally photographed at predetermined time intervals (18, 24, 48 h and 7 days) for determining the inhibition zones by measuring the halos with the software Image J. Additionally, the MOF precursors (AgNO_3_ and BDC) were used as controls in proportional concentrations of the bulk material (17 and 9 ppm for 20 ppm of MOF, respectively). All experiments were repeated at least three times [47].

#### 2.4.3. Suspension Assay against Planktonic Bacteria

Diverse concentrations of AgBDC MOF powder (1, 2.5, 5, 10, 20, 50, 100 and 200 ppm) were suspended over 2.25 mL of the previously mentioned 10^6^ bacteria mL^−1^ *E. coli* inoculums on a disposable 24-well plate for 18 h at 37 °C. Once again, AgNO_3_ and BDC ligands were used as controls in proportional concentrations (i.e., for 20 ppm of MOF, 9 and 17 ppm of ligand and AgNO_3_, respectively). These inoculums with MOF suspensions were used for determining planktonic bacteria viability by optical density (OD_600_), colony counting (colony forming units, CFU) and enzymatic activity (fluorescein diacetate, FDA) [40,48].

The optical density was determined with aliquots of the suspensions of the controls and the sample in contact with the bacteria, measuring the absorbance (OD_600_; λ = 600 nm) in a UV-1800 spectrophotometer. Results are represented as % of inhibition. The colony count was performed from the aliquots (of both planktonic and sessile bacteria) that were placed in sterile 96-well microtiter plates in 10-fold serial dilutions in PBS. The amount of 10 μL were inoculated into Petri dishes (Ø = 9 cm), containing NB agar composed of glucose, yeast extract and tryptone. Finally, they were incubated at 37 °C for 24 h, and bacterial growth was quantified using a CL-1110 counting instrument (Acequilabs, Spain). All experiments were carried out in duplicate or triplicate and in accordance with ISO 22196. Results are presented as the ratio of the Log_10_ CFU·mL^−1^ of the sample vs. the control.

Finally, the enzymatic activity was evaluated, determined using FDA, a nonfluorescent compound hydrolyzed by esterases in fully functional cells to a green, fluorescent compound, fluorescein. For doing so, 5 μL of a prepared FDA solution at 2 mg·mL^−1^ was mixed in dimethyl sulfoxide (DMSO) along with 195 μL of the sample bacteria suspension in a 96-well plate. The plate was incubated at 25 °C for 30 min, with readings performed every 5 min (exc. 485 nm; em. 528 nm) using an Fluoroskan Acent FL model fluorometer, (Thermo Scientific brand, Waltham, MA, USA). The possible interference of the culture medium and the MOF with the fluorescence measurement was determined before checking the activity. Each sample was measured four times, and the results are presented as reduction %.

#### 2.4.4. Thin-Film Assay against Sessile *E. coli* Biofilm

AgBDC thin films (at 100 ppm as final MOF concentration) along with their controls (glass discs without material) were put in contact with 2.5 mL of the previously mentioned *E. coli* inoculums in 24 well-plates at 37 °C for 18 h and 7 days, displacing the discs with the active layer facing up and while remaining at the bottom without floating. Supernatant liquid was used for OD_600_ and FDA measurement, analyzing the bacteria colony forming cell (CFC) considered to be *planktonic* bacteria, and the cells removed from the biofilm formed over the cover glasses were considered to be the sessile type. For the removal of the sessile bacteria from the cover glasses, a previously reported procedure was followed [49].

Furthermore, because of the potential antiadherent and antibacterial properties of the AgBDC thin films, their activity was evaluated via the confocal laser scanning microscopy (CLSM) technique. After incubation of the cover glasses with the mentioned *E. coli* inoculum during 90 min and 48 h, the discs were washed three times with sterile PBS, staining them with 3 µL·mL^−1^ of Live/Dead^®^ Bacterial viability kit (BacklightTM; which contains a mixture of Syto 9 and propidium iodide, PI). Additionally, 5 µL·mL^−1^ of calcofluor solution was added to label “in blue” the mucopolysaccharides of the biofilm (extracellular matrix), which specifically determine the biofilm formation. Both reactants were incubated for 15 min at RT. Confocal images were obtained using an Olympus FV1200 (Tokyo, Japan) confocal microscope, collecting 8 photographs (60× magnification) of each sample. This technique identifies the bacterial viability through the integrity of the cell membrane: viable bacteria emit *green* fluorescence due to Syto 9 fluorochrome (ƛex 480 nm/ƛem 500 nm), while nonviable bacteria emit *red* fluorescence due to PI (ƛex 490 nm/ƛem 635 nm). In addition, the formation of biofilm is revealed in blue (ƛex 350 nm/ƛem 432 nm) [50]. All images are representative of three independent experiments.

Antifouling capacity of the materials was likewise assessed using an SEM system (DSM-950 Zeiss, Oberkochen, Germany). For SEM micrographs, samples were washed twice with PBS to remove nonattached bacteria from the surface (AgBDC thin film and control glass disc). Next, the remaining bacteria were fixed in glutaraldehyde (2.5%) for 2 h and then dehydrated with gradient ethanol (30–100%) and acetone. Each sample was dried with hexamethyldisilazane for 15 min prior to sputter coating with gold for SEM observation. All images are representative of three independent experiments.

## 3. Results and Discussion

### 3.1. Synthesis and Characterization

**Figure 1:**
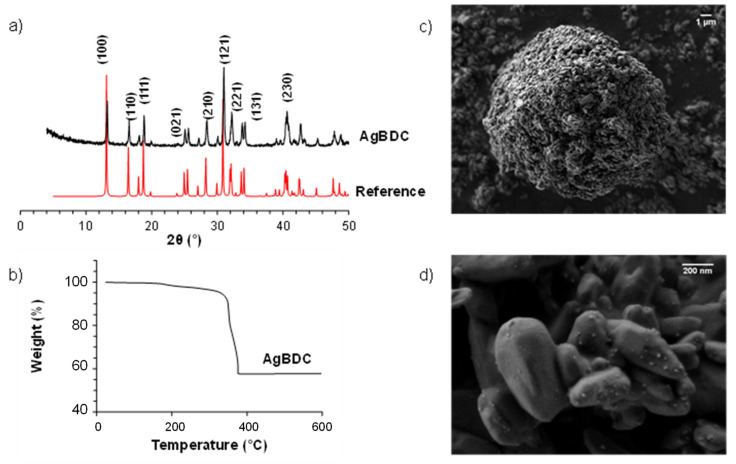
Characterization of synthesized AgBDC MOF. (**a**) XRPD patterns of as-synthesized AgBDC (up) compared to the simulated reference (down). (**b**) TGA and (**c**,**d**) SEM micrographs of AgBDC samples. Scale bar: 1 µm (**c**) and 200 nm (**d**).

The AgBDC MOF was first reported by Sun et al. [41]. Nevertheless, in this work, the synthetic procedure was optimized (see Section 2) in order to avoid solvent heating, reducing the reaction time (1 vs. 7 days) and upscaling the AgBDC production per reaction batch (250 vs. 10 mg) [51]. Thus, this simpler and faster method procured a grey-brownish crystalline powder that perfectly matched the structure of the previously reported silver terephthalate coordination polymer AgBDC (see X-ray diffraction patterns–XRPD in Figure 1) [41].

Further analyses of AgBDC indicated that the material exhibited a slight weight loss up to ~300 °C (see TGA, Figure 1). Afterwards, the decomposition profile showed an important weight loss of ~42% corresponding to the departure of the organic linker. The remaining solid residue, identified as Ag° (Figure S2), showed a slightly lower Ag content (53 wt.%) in comparison with the theoretical formula of AgBDC (56 wt.% for AgC_4_H_2_O_2_) [45,46,51,52]. This divergence might arise due to the presence of an extra ligand, presumably attached to Ag at the boundaries of crystallites, covering the Ag centers from water molecules, which is in line with TGA results of a hydrophobic material [53]. Despite this variation, the TGA value was in agreement with the amount of silver determined through inductively coupled plasma optical emission spectroscopy (ICP-OES), 52.3 ± 2.1 wt.%. Field emission gun scanning electron microscopy (FEG-SEM) images showed submicrometric and slightly defined grain-shaped crystals (between 250 ± 210 nm) that tend to agglomerate in bigger particles (10 ± 0.1 µm; *n* ~200 particles were measured, see Figure 1). Even direct comparison with original synthesis is not possible, as no micrographs were presented; a recent work with a synthesis based on ammonia procured similar AgBDC crystals in shape and size [54].

### 3.2. AgBDC Stability Suspended in Biological Media

Bearing in mind the biocide application of AgBDC, it is crucial to investigate the maintenance of the MOF physicochemical features under relevant environments: mainly, its structural and colloidal stability in contact with physiological media such as deionized water or culture media (NB). These studies will shed light on its biological activity and suitability for the desired antimicrobial purposes. As can be seen in the XRPD patterns (see Supplementary Materials, Figure S3), AgBDC preserved its crystalline structure after 18 h at 37 °C in water or NB, the required conditions for the biological experiments. Regarding the evolution of the hydrodynamic particle diameter, dynamic light scattering (DLS) measurements were performed (disclosed in Table 1). In aqueous solution, a monodisperse submicrometric size (300 ± 130 nm) was observed and was in good agreement with the obtained dimensions from FEG-SEM micrographs (250 ± 210 nm). In more complex media (NB bacterial broth), the presence of other components such proteins, vitamins, etc., seems to partially stabilize the size, reaching smaller particle average in comparison with water (100 ± 70 vs. 300 ± 130 nm), which is associated with higher polydispersity indexes (PdI; 0.45 vs. 0.30). In terms of the surface charge, strong negative values were obtained (−50 ± 5 and −30 ± 1 mV) for both media, probably because of the presence of carboxylate moieties on the surface. The ζ-potential decreased in the case of NB growth due to the influence of the other medium compounds, which is also in agreement with the observed size variation [47]. As reported by Moore et al. [48], when nanoparticles are dispersed in culture media, proteins are strongly adsorbed on the surface (forming the so-called protein corona), which affects the surface charge distribution and, subsequently, the colloidal stability. This effect has been also observed in other MOF formulations, where the control of their biodistribution with diverse biopolymers was performed [49]. Moreover, these analyses were monitored after 18 h of contact time (see Supplementary Materials, Figure S4), exhibiting high-quality stability values, indicating that AgBDC will be available in the suspension for bacteria inhibition.

### 3.3. AgBDC Thin-Film Stability in Biological Media

More interesting and challenging than the antimicrobial treatment of planktonic bacteria (in suspension) is the development of antiadherent and biocide surfaces that could both prevent and treat bacterial biofouling. This is the reason why AgBDC MOF was deposited by spin coating on glass disc substrates to form an active thin-film coating (see Section 2 and thin-film images in Supplementary Materials, Figure S5). Here, DLS measurements were also used to assess the potential detachment of the AgBDC particles from the thin film after the contact with the NB for 7 days by determining the particle size in the supernatant NB medium (see Supplementary Materials, Figure S6). As the diameter of the particles detected was around 10 ± 8 nm, which is an order of magnitude below the AgBDC MOF crystal size, it was concluded that the DLS just quantified the protein size from the NB (i.e., a blank experiment of NB without any MOF had an average size of 5 ± 3 nm). This size change over the time could be associated to its agglomeration and potential variation of its conformation [39]. Objects of 100–400 nm were not detected in NB, ruling out the release of the AgBDC particles and, consequently, concluding that AgBDC was strongly attached to the substrate, which remained available in the thin film during long periods of time (7 days).

Despite the fact that no AgBDC particles were released into the medium, the MOF constituents (i.e., Ag^+^ and BDC) could still be released to the biological media by its decomposition. Bearing this in mind, researchers more frequently determine the silver ions delivery from Ag-based MOFs [50,51,55]. In this work, the supernatant in contact with the AgBDC thin-film coating was analyzed by quantifying not only the released Ag+ (by ICP-OES), but also the organic 1,4-benzene dicarboxylate (BDC) ligand (via HPLC).

**Figure 2:**
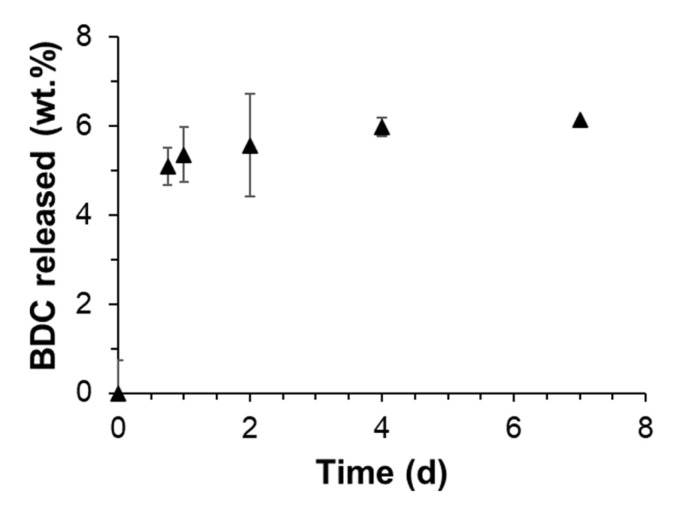
Degradation kinetics of AgBDC thin-film coating incubated in NB at 37 °C.

It was observed that after the first 24 h, a fast BDC release (5 wt.% delivered from the total in the coating; Figure 2) was obtained with NB at 37 °C. After that time, it steadily reached a plateau of ~6 wt.% at 7 days. Considering the scarce amount of released BDC to the media after 7 days and the burst initial trend, it is believed that it corresponds to the excess of ligand attached to the boundaries of the AgBDC structure, already identified by TGA and ICP. On top of that, no release of Ag+ was detected during this period, being below the limit of detection (LOD; <0.05 ppm). Thus, due to the observed experimental findings, it could be concluded that the AgBDC coating was highly stable under the biocide experimental conditions, remaining strongly attached and available during the whole 7-day test. The high stability of the conformed AgBDC coating is in line with previously reported results for the amino-substituted AgBDC-NH_2_ synthesized on top of a thin-film membrane, where just 0.4 ppm of Ag was released in 5 days under flux [15]. 

### 3.4. Determination of AgBDC Bactericidal Activity

**Figure 3:**
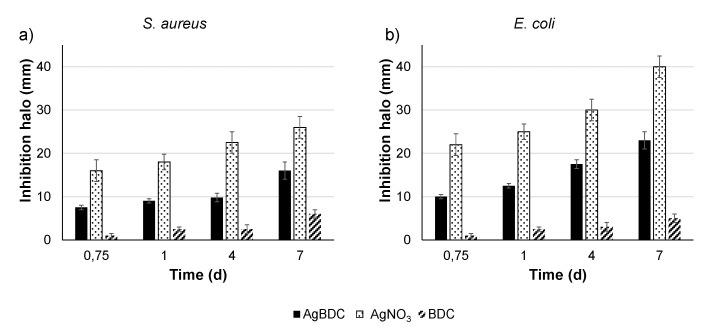
Inhibition halo (mm; determined by Image J) of AgBDC against: (**a**) *S. aureus* and (**b**) *E. coli*.

As a first attempt, the AgBDC biocide character against *S. aureus* and *E. coli* was investigated by an agar diffusion test (in solid shape), determining the inhibition halo produced by the presence of AgBDC together with the equivalent amount for the tested concentration of AgNO_3_ and BDC, as main precursors. Representative images of the halo evolution over time are in SI (see Figure S8), while their quantification is presented in Figure 3. Remarkably, AgBDC had the potential to treat both *S. aureus* and *E. coli* infections, showing a higher effect on *E. coli.* A similar trend was reported for the aminated analogue AgBDC-NH_2_ deposited on membranes, observing a slighter inhibition effect when incubated with *S. aureus* compared to *E. coli* [17]. For both Gram (+) and Gram (−) bacteria, the inhibition halo formed by the powdered AgBDC was not only maintained, but also boosted during the 7 days of experiment. As mentioned in the state of the article, when studying other Ag-based MOFs inhibition, most agar tests consider the measurement of the area just in a single time (overnight or 24 h of incubation); thus, a direct comparison is not straight forward [17,20,21]. An example of a long time examination was reported for the silver-triazole coordination polymer Ag-TAZ [56]. When exposed also in powder form against *Anabaena* and *Synechoccus* cyanobacteria, the inhibition zone remained constant with time. This emphasizes the appealing biocidal activity of AgBDC that is not only kept over time, but also is able to prolong and boost its performance.

In the particular case of the AgNO_3_ control, a higher inhibition compared to the whole MOF was exhibited. This behavior could be explained by the greater availability of cation Ag^+^ in the Petri dish due to the higher solubility of the silver salt compared to the MOF in water and agar [57]. This is in conformity with the exceptional stability of AgBDC, determined in the previous section. On the other side, the BDC ligand performed a lower inhibition compared to the MOF (i.e., after 1 day, the BDC diameter was 2.5 mm vs. 9.0 mm for *S. aureus* and 12.5 mm for *E. coli* in the case of AgBDC). Hence, the BDC ligand displayed some biocide characteristics against both bacterial strains; therefore, the AgBDC MOF bioactivity must arise from the silver present in its structure and the potential interaction of the MOF crystals with the bacteria membrane, as no release of this cation was detected under these working conditions.

However, the organic ligand showed some antibacterial activity (85 vs. 65% bacteria inhibition at 20 ppm from AgNO3 and BDC, respectively; Table S1), thus being also beneficial for the resulting bactericidal activity of the AgBDC MOF.

**Figure 4:**
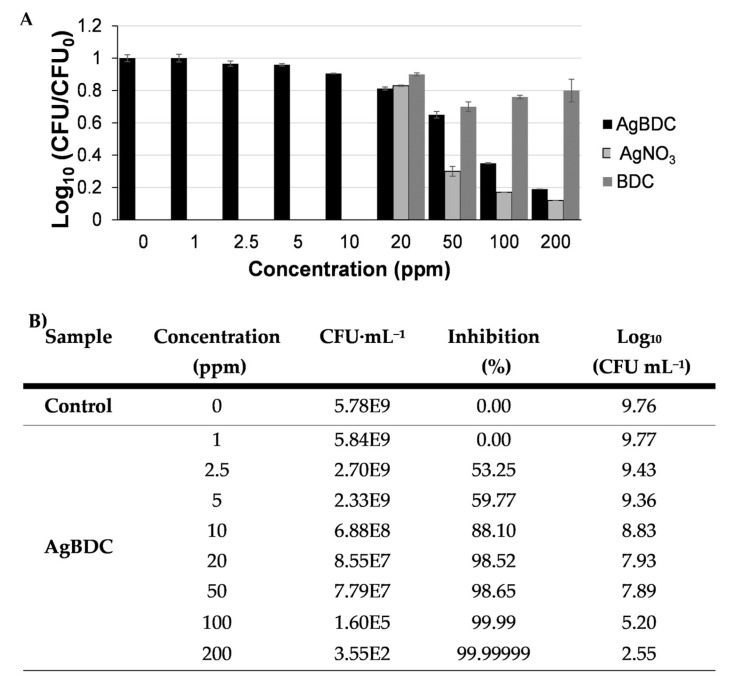
(**A**) *E. coli* CFU·mL^−1^ (represented as the logarithmic ratio, with C0 being the CFU·mL^−1^ of the positive control for better comparison) in contact with AgBDC suspension after 18 h of incubation. (**B**) Determination of planktonic *E. coli* bacterial viability by plate count after 18 h in contact with the AgBDC suspension: the correlation between CFU·mL^−1^, Log_10_ and inhibition %.

As agar diffusion assays indicated that AgBDC had a higher effect against *E. coli* (see Figure 3), this strain was selected for the following bactericidal experiments. Determination of the minimum inhibitory concentration (MIC) of AgBDC suspension against planktonic *E. coli* was determined by counting the CFU against growing concentrations of AgBDC suspensions (Figure 4). From these data, an MIC within the 100–200 ppm range (corresponding to 50–100 ppm of Ag^+^ alone) could be estimated. Despite this being a relatively high Ag^+^ concentration compared to other Ag-based MOFs (see SI, Table S2), it should be highlighted that the elevated stability of AgBDC under biological media might favor: (i) a minimum Ag^+^ leaching to the NB over extended periods of time, and (ii) the lower bactericidal or extra inhibition effect coming from the ligand release (see SI, Table S1). In contrast with those other reported structures, whose activity arises from Ag^+^ diffusion through the bacteria membrane and, subsequently, reactive oxygen species (ROS) generation [8,58,59], AgBDC particles might be the principal trigger for the observed *E. coli* bacteria impact. In this sense, complementary bacterial viability assays were performed, such as determination of OD_600_ and the reduction of enzymatic activity by fluorescent fluorescein diacetate (FDA). These outcomes showed similar CFU rates as previously obtained, with a clear dependent-concentration increase in the bacteria inhibition produced by AgBDC, which was similar to the AgNO_3_ control effect (see SI, Figure S9).

Because the main interest of this work is to investigate the potential inhibition of challenging biofilms, as a step further, AgBDC thin-film coatings were incubated with *E. coli* inoculum in order to evaluate its capability to face the biofouling (see, in SI, Figure S10). For the study, both types of bacteria, the ones in suspension (planktonic bacteria) together with the biofilm-forming type detached from the surface (sessile bacteria), were quantified (see Table 2). AgBDC thin-film coatings (100 ppm under the experiment conditions) exhibited a strong biocide effect, even after 18 h of incubation, providing a fruitful activity against planktonic *E. coli* with a 99.9999% inhibition of the culture growth. A similar powerful effect was observed versus sessile *E. coli*, as viable CFU in contact with AgBDC is three orders of magnitude lower than the ones detached from a control glass disc (9.17 × 10^1^ vs. 5.72 × 10^4^ CFU·mL^−1^, respectively). In other words, a 99.84% viable biofilm inhibition was achieved.

Considering that the CFU logarithmic ratio (average value of ~0.4 for both suspension and biofilm viable bacteria) in contact with AgBDC thin-film coating (see SI, Figure S11) exhibited the same range as for AgBDC suspensions at 100 ppm (see Figure 4), this could imply that AgBDC biocide activity is not diminished after the thin-film conformation. Moreover, an important bacterial inhibition (>80%) of the planktonic *E. coli* obtained was also confirmed in the supernatant after the MOF contact via OD_600_, observing a decrease in their enzymatic activity (~70% reduction of the FDA fluorescent emission; see SI, Figure S12).

As was previously observed with the agar diffusion test, the antimicrobial effect of AgBDC coating was kept after 7 days of incubation with an additional inhibition boost for both sessile and planktonic *E. coli.* Even other Ag-MOF coatings have previously been considered as potential treatments for bacterial biofouling; prior literature often assesses the biocide effect of the coatings qualitatively by means of visualizing the surface by confocal microscopy just after a brief incubation period (from 1 to 24 h) [17,19,22]. However, it is highly recommendable to extend the study timeline for its approval, as has been conducted in this publication. In addition, for an effective quantification of the system, it is necessary to ensure the biocide activity of the coating during the complete lifetime of the MOF device (e.g., water filtration membranes, wound healing patches, coatings) [23,60].

**Figure 5:**
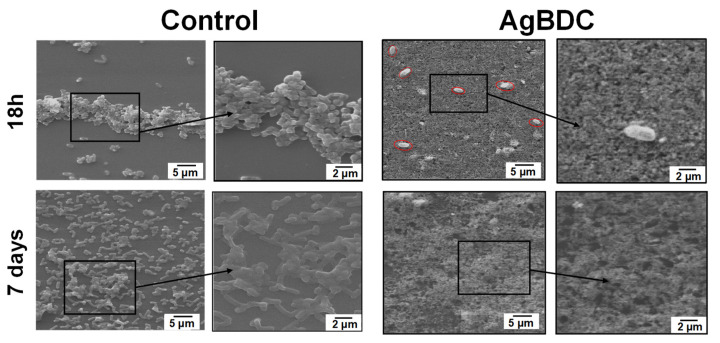
SEM images of *E. coli* biofilm on cover glass surface: AgBDC coating (**right**) after biofilm growth for 18 h (**up**) and 7 days (**down**) compared with the control (**left**); zoom images (2 µm scale) are highlighted (squared, **right**).

As important as the antimicrobial character of the AgBDC coating for the inhibition of challenging *E. coli* biofilm are its antiadherent properties. Observation of the biofilm growth with the assistance of SEM (see Figure 5) reveals attached *E. coli* bacteria to the control glass substrate, which starts to agglomerate in a colony form after 18 h. In contrast, in the case of the AgBDC coatings, very few and isolated *E. coli* bacteria are spotted (marked in Figure 5 for better visualization). Under higher magnification, the attached *E. coli* exhibits a rough membrane surface compared to the control. This alteration could be due to the potential stress of the bacteria in contact with the AgBDC coating, leading to bacterial death, as previously stated by other authors [19,21,24].

Monitoring the *E. coli* biofilm growth in the control after 7 days revealed a higher amount of bacteria colonization on the surface. Even more, with higher magnification, the formation of extracellular polymeric substances (EPS) binding along with the microbial aggregation was confirmed. Conversely, no sight of *E. coli* was found over the AgBDC coating, verifying the long-time biocide and antifouling performance of this AgBDC coating.

**Figure 6:**
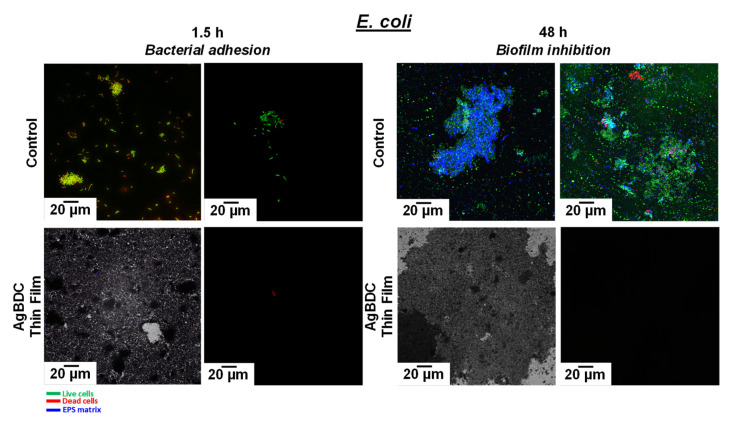
Fluorescence LIVE/DEAD confocal images of sessile *E. coli* biofilm (bottom right) and transmission mode (bottom left) on cover glass surface were taken after 1.5 h («bacterial adhesion») and 48 h («biofilm growth») of contact with AgBDC thin films (100 ppm; bottom). The MOF absence was used as control (top), highlighting with specific staining the bacterial viability (viable: green; dead: red) and the biofilm formation (blue). The scale bar corresponds to 20 μm. All the images were taken at 63×.

In addition, the effectiveness of AgBDC coating preventing bacterial adhesion (antifouling) and its antimicrobial capacity were also evaluated by confocal laser scanning microscopy (CLSM). As a distinctive feature of the CLSM analysis presented by other researchers when analyzing MOFs as bactericidal agent, here, the use of calcofluor for specifically staining the EPS matrix of a biofilm along with the well-known fluorochromes Syto 9 and PI (Live/Dead kits) is originally reported [61,62]. Even if the determination of bacteria viability (red-green) is vital for analyzing the MOF bactericide effect, it is also required to assess the MOF biofilm inhibition ability (i.e., preventing EPS formation or the dispersion of the mucopolysaccharides after biofilm destruction), which appears in blue in the CLSM micrographs. Representative images of the AgBDC effect on *E. coli* biofilm are depicted in Figure 6. After 90 min, live bacteria colonies appeared in the control disc (in green), starting their adhesion to the substrate. After this time, almost no attached bacteria cells were appreciated to the AgBDC coating, neither in the confocal (bottom right) nor in the transmission mode (bottom left). Therefore, these findings determined the capability of AgBDC MOF to restrain *E. coli* initial bacterial adhesion. Furthermore, it caused bacterial death to those in contact, as seen in red-stained bacteria from the CLSM image, which is in agreement with the CFU analysis.

Remarkably, the antifouling properties of the AgBDC coating were sustained even after 48 h of incubation. At this time, the untreated control sample displayed the typical structure of a healthy *E. coli* biofilm composed of colony live bacteria (green) integrated in a protective mucopolysaccharide matrix (blue). In contrast, the MOF-coated substrate led to the absence of *E. coli* bacteria attached to the surface, not even observing any killed bacteria with the red fluorescent staining. In consequence, as no bacteria were adhered, no biofilm (blue-stained mucopolysaccharides) proliferation was promoted. In other words, these outcomes indicated that AgBDC not only prevented bacterial adhesion, but also their biofilm formation.

These qualitative results have uniformity with the observations of the AgBDC coating obtained by SEM, which, along with the quantitative determination of bacterial viability, highlight the strong potential of the AgBDC MOF for the antimicrobial treatment of *E. coli*, showing a combined antifouling and biocide effect during long time periods (7 days). The capacity of AgBDC to prevent initial bacterial adhesion is a remarkable feature not reported so far for other Ag-based MOFs against biofilm formation (see Table S2) and scarcely mentioned for other antimicrobial MOFs (pure or as composites) [39,63].

## 4. Conclusions

The up-scaled protocol here described for the synthesis of AgBDC is a simple and quick methodology for the production of submicronic crystals (~250 nm), which could be easily conformed in thin-film coatings. AgBDC exhibited great (colloidal, structural and chemical) stability in water and biological culture broth media up to 7 days, with a relevant biocide activity against *S. aureus* and *E. coli* strains (MIC*_E. coli_* ~ 100 ppm).

Further, AgBDC thin-film coatings originally presented an important antifouling effect, preventing not only the *E. coli* bacteria initial adhesion to substrates but also inhibiting the proliferation of challenging biofilms. The AgBDC coatings were able to inhibit > 99% of bacterial viability, both in planktonic and sessile state. Altogether, its antiadherent and biocide properties make AgBDC a promising multifunctional material for the development of coatings for the attainment of bacteria-free surfaces, which is very useful in relevant applications (e.g., disease spread control, implants, food packaging, water treatment, heat exchange and biocorrosion).

## Figures and Tables

**Table 1:** Colloidal characterization of AgBDC suspensions in water or NB at 37 °C.

Media	Hydrodynamic Particle Size (nm)	Polydispersity Index (PdI)	ζ-Potential (mV)
H_2_O	300 ± 130	0.30 ± 0.03	−50 ± 5
NB	100 ± 70	0.45 ± 0.01	−30 ± 1

**Table 2:** Determination of planktonic and sessile *E. coli* bacterial viability by plate counting after 18 h (0.75 days in dark) or 7 days contact with the different proposed materials, expressed as CFU·mL^−1^, inhibition % (with respect to the control CFU·mL^−1^) and Log_10_ (CFU·mL^−1^).

		*Planktonic* Bacteria			*Sessile* Bacteria		
Sample	Contact Time	CFU·mL^−1^	Inhibition (%)	Log_10_ (CFU·mL^−1^)	CFU·mL^−1^	Inhibition (%)	Log_10_ (CFU·mL^−1^)
Control	<1 day	2.70 × 10^9^	0.0000	9.43	5.72 × 10^4^	0.0000	4.76
AgBDC		1.57 × 10^3^	99.9999	3.20	9.17 × 10^1^	99.84	1.96
Control	7 days	2.42 × 10^11^	0.0000	11.38	1.53 × 10^10^	0.0000	10.18
AgBDC		3.20 × 10^4^	99.99999	4.51	3.38 × 10^3^	99.9999	3.53

## Data Availability

Not applicable.

## Supplementary Materials

The following supporting information can be downloaded at: https://www.mdpi.com/article/10.3390/pharmaceutics15010301/s1. In the supporting information are additional results of material characterization (FTIR, Figure S1), stability (XRD, DLS, HPLC; Figures S2–S7, Table S1) and bactericidal activity of the AgBDC suspensions and thin-film coatings (inhibition halo, CFU, OD600, FDA; Figures S8–S12, Tables S1 and S2). A table comparing the present work with previous state of the art of biocide Ag-based MOFs is also included.
