# Supplementary material for: Antiadherent AgBDC Metal–Organic Framework Coating for Escherichia coli Biofilm Inhibition
Source: Pharmaceutics. 2023 Jan 16;15(1):301. doi: 10.3390/pharmaceutics15010301 (PMC9866433; doi:10.3390/pharmaceutics15010301)
Supplement: Supplementary file 1 [file pharmaceutics-15-00301-s001.zip › pharmaceutics-2088990-supplementary.pdf]

pharmaceutics

MDPI

Supporting Information
Antiadherent AgBDC Metal-Organic Framework Coating for *Escherichia coli* Biofilm Inhibition

1. Material characterization

AgBDC: Brown solid, yield 43 %. FTIR (ν, cm^{-1}): 740, 528, 454 (O-Ag-O), 670 – 1228 (C-H), 1492 (COO^-). PXRD (2θ $(°)_{[h\ k\ l]}$): 13.2 $_{[1\ 0\ 0]}$, 16.6 $_{[1\ 1\ 0]}$, 18.8 $_{[1\ 1\ -1]}$, 25.1 $_{[0\ 2\ 1]}$, 25.6 $_{[1\ 1\ 1]}$, 28.3 $_{[2\ 1\ 0]}$, 30.90 $_{[1\ 2\ 1]}$, 32. $_{[2\ 2\ -1]}$, 34.2 $_{[1\ 3\ -1]}$, 40.6 $_{[2\ 3\ 0]}$, 42.6 $_{[1\ 4\ 0]}$. TGA (wt.%): 56%.

FTIR analysis confirmed the formation of the bond between silver and the carboxylates with the vibrational band ν(C-O) at 1492 cm^{-1} Similarly, vibrational bands Ag-O at 740 cm^{-1} and the bending vibrations of the Ag-O fragment at 528 and 448 cm^{-1} were also observed (**Figure S1**). All these results are in agreement with preceding stated characterization [41].

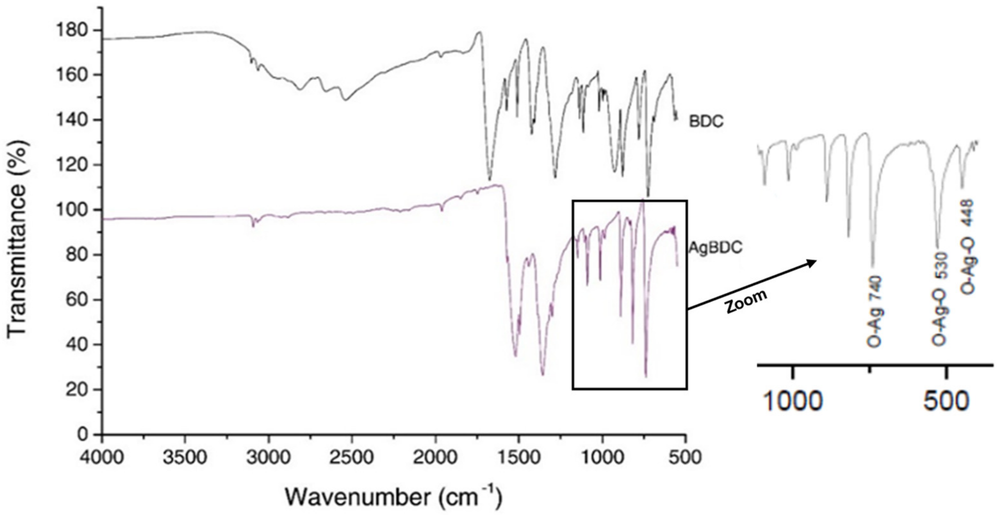

Figure S1. FTIR spectra of ligand BDC (up) and AgBDC MOF (down).

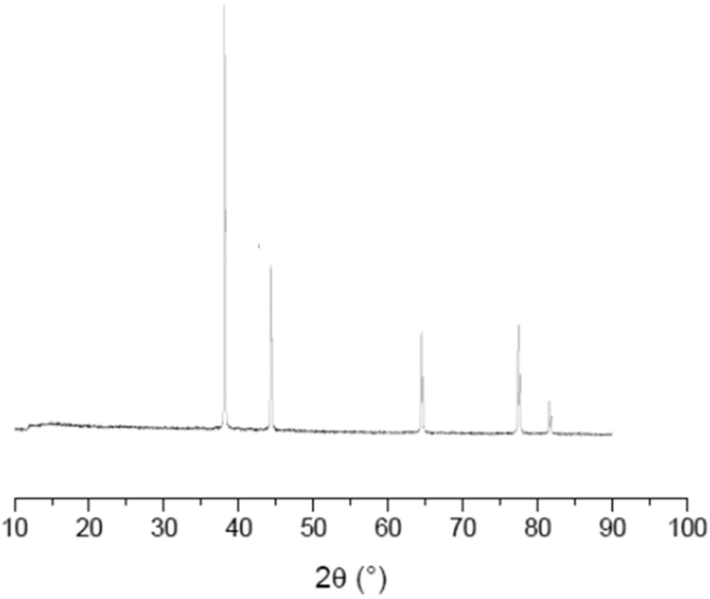

Figure S2. XRPD patterns of the remaining solid residue of AgBDC after TGA measurement.

2. AgBDC stability suspended in biological media

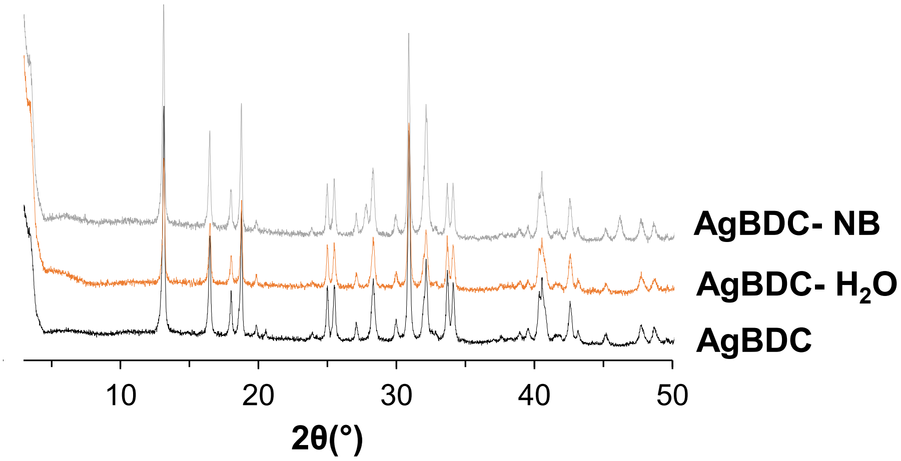

Figure S3. XRPD patterns of AgBDC as synthesized (bottom, black) and after 18 h incubation at 37 °C in DI water (medium, orange) and NB (top, grey).

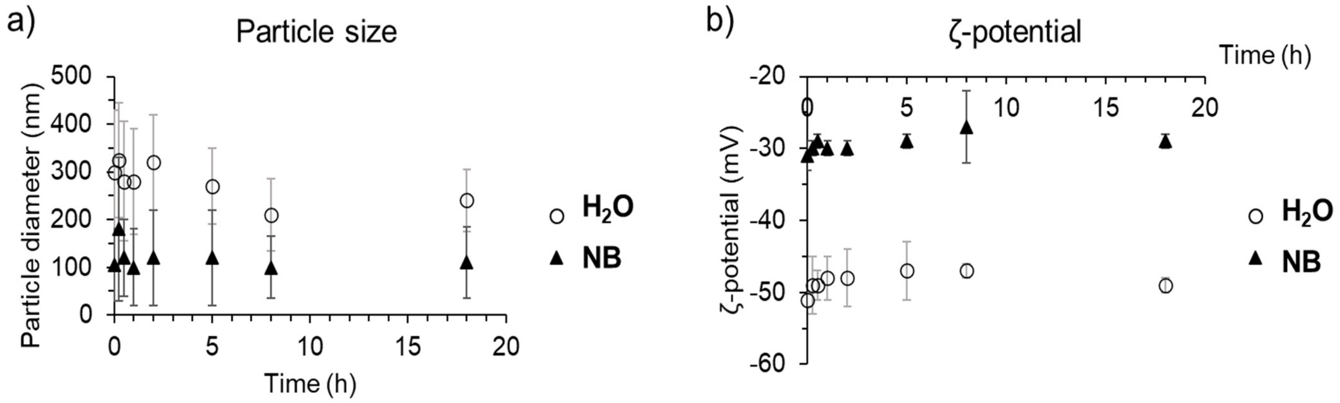

Figure S4. Analysis of AgBDC (a) particle size and (b) colloidal stability with time at 37°C in suspension with water (circles) and NB (triangles).

3. AgBDC thin film stability in biological media

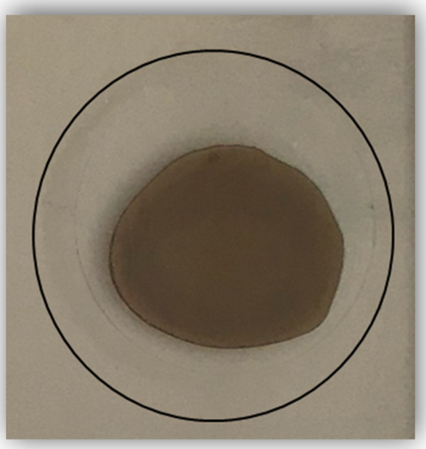

Figure S5. Glass disc with an AgBDC thin film coating.

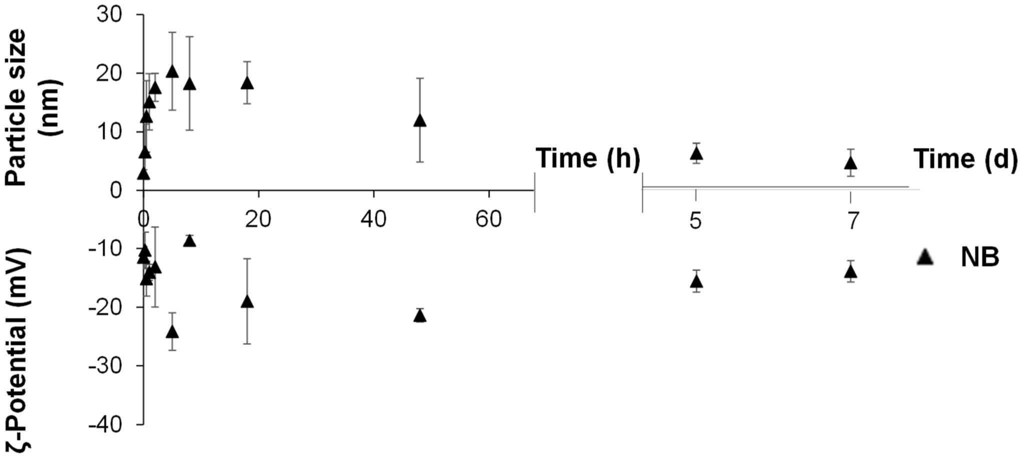

Figure S6. Particle size and ζ-potential as a function of the time of the supernatant NB medium in contact with the thin film coating of AgBDC incubated at 37 °C.

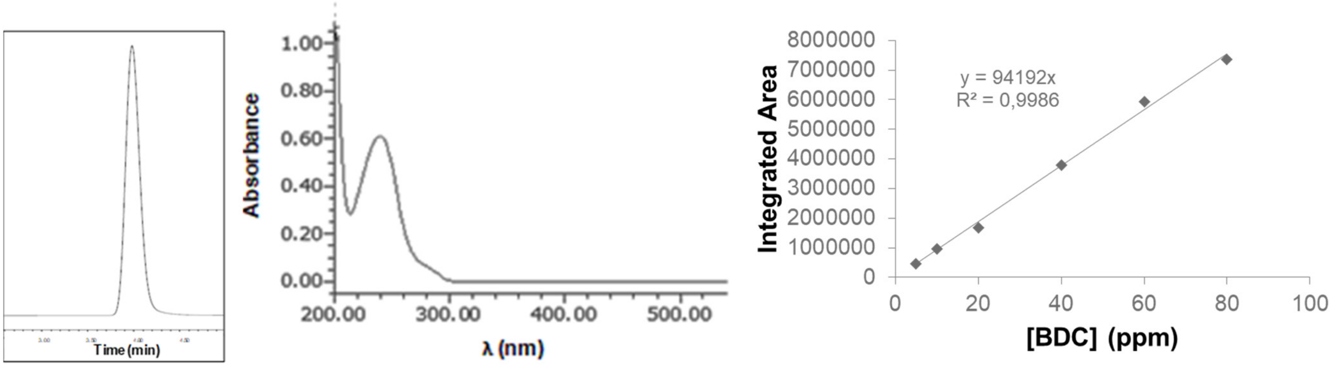

Figure S7. Chromatogram of BDC, UV-vis spectrum and calibration plot of standards by HPLC method.

4. Determination of AgBDC bactericidal activity

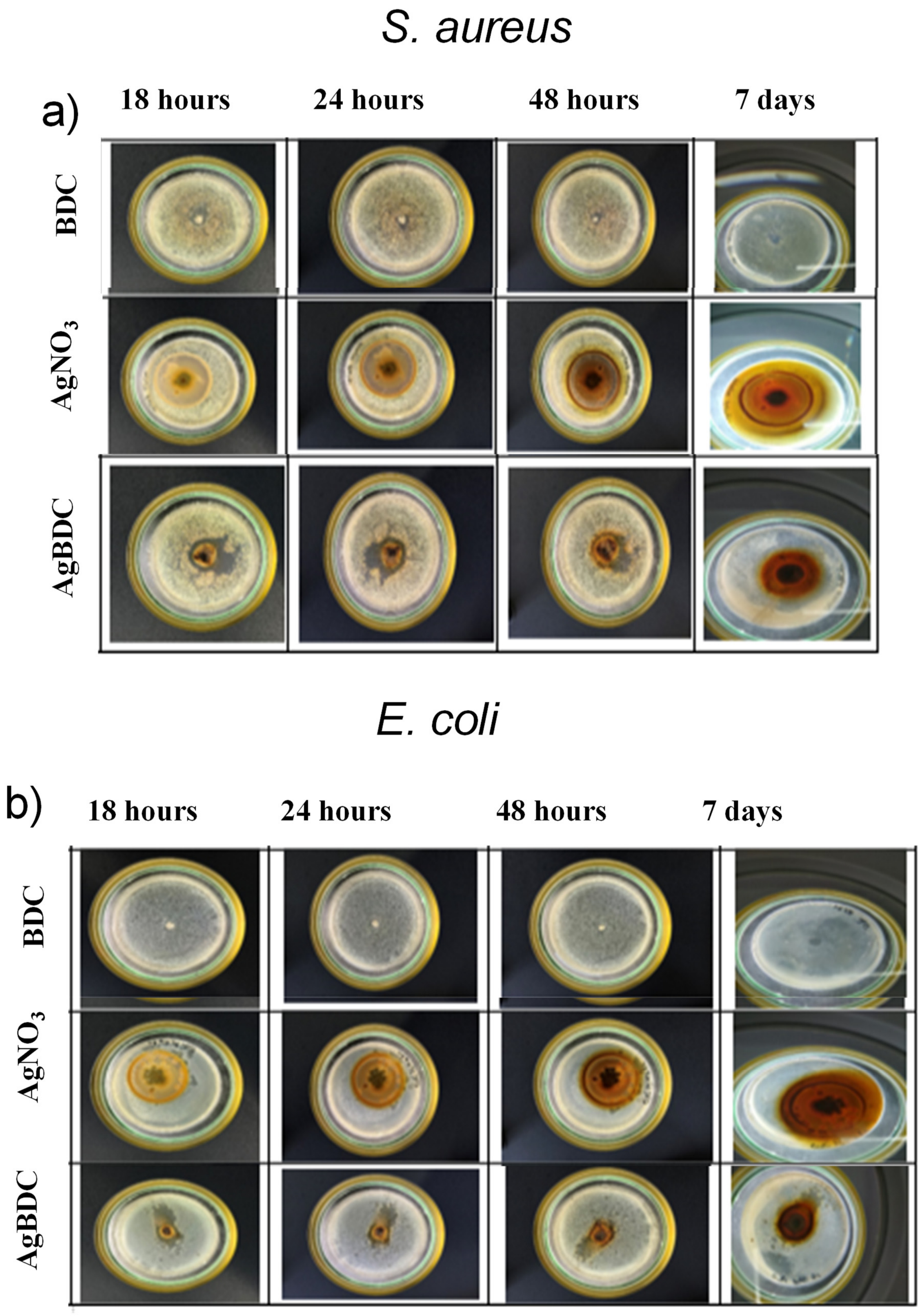

Figure S8. Representative images of inhibition halo with time of BDC, $AgNO_3$ and AgBDC MOF against (a) *S. aureus* and (b) *E. coli*.

Table S1. Determination of planktonic *E. coli* bacterial viability by plate count after 18 h in contact with the controls $AgNO_3$ and BDC in suspension, expressed as CFU·mL^{-1}, inhibition% (with respect to the control CFU·mL^{-1}) and Log10(CFU·mL^{-1}).

Sample	Concentration (ppm)	CFU·mL^{-1}	Inhibition (%)	Log_{10} (CFU · mL^{-1})
**Control**	0	5.78E+09	0.00	9.76
**$AgNO_3$**	20	8.97E+08	84.48	0.83
	50	3.41E+04	99.999	0.30
	100	1,17E+02	99.99999	0.17
	200	3.58E+00	99.9999999	0.12
**BDC**	20	2.02E+09	65.13	9.30
	50	1.63E+08	97.17	8.21
	100	3.72E+08	93.57	8.57
	200	6.21E+08	89.26	8.79

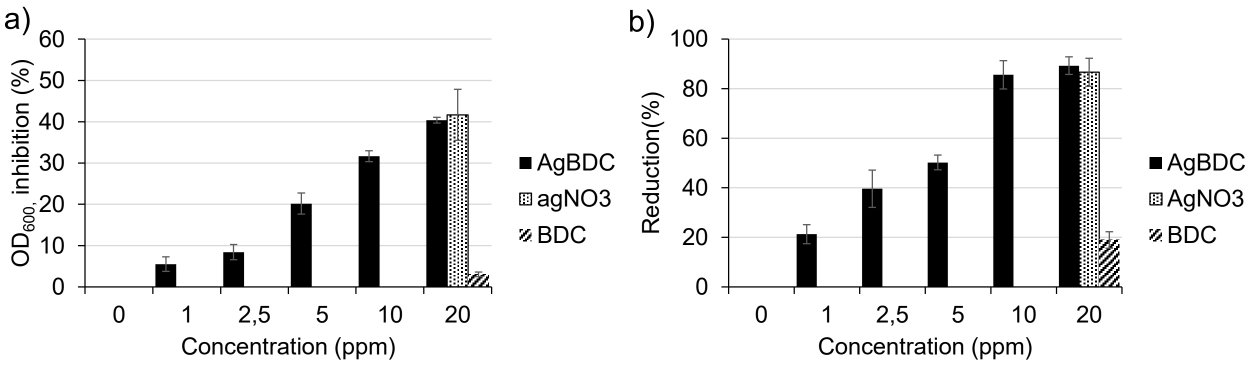

Figure S9. AgBDC bactericide activity in suspension against *E. coli* after 18 h incubation at 37 °C. In (a) bacterial viability inhibition determined by OD_{600} and (b) enzymatic activity reduction determined from FDA fluorescent emission (λ_{ex}485 nm; λ_{em} 538 nm).

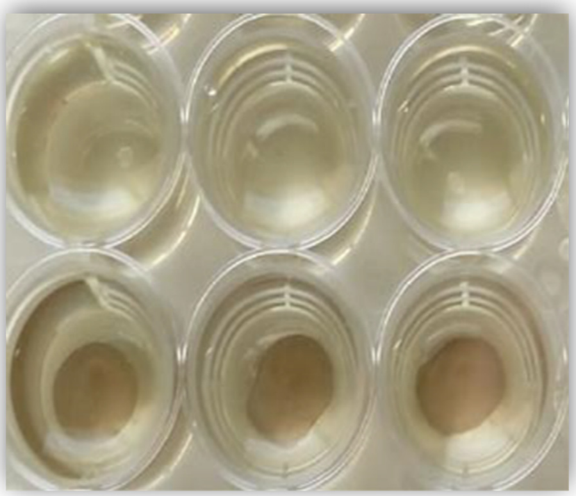

Figure S10. Control glass discs and AgBDC thin film coating with *E. coli* inoculum in a 24-well plates.

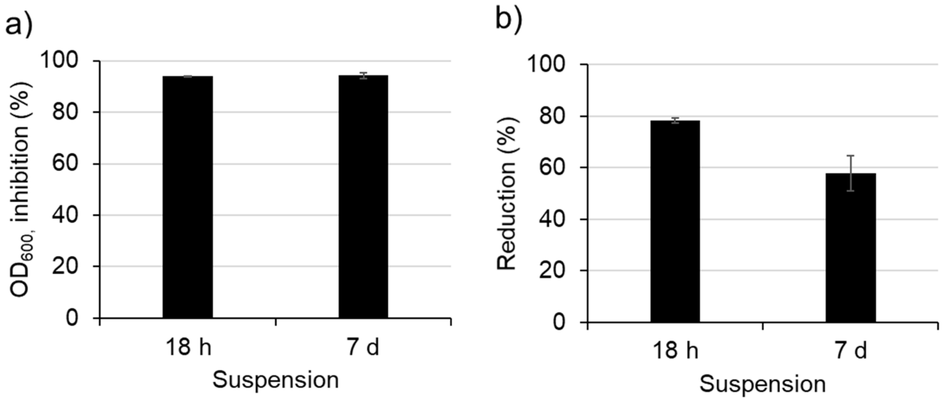

Figure S11. Colony forming units·mL-1 (represented as the logarithm ratio, being C_0 the CFU·mL1 of the positive control for better comparison) of the *E. coli* suspension (planktonic bacteria) and *E. coli* biofilm detached from the surface of the AgBDC thin film coating, after (a) 18 h of incubation and (b) 7 days of incubation at 37 °C.

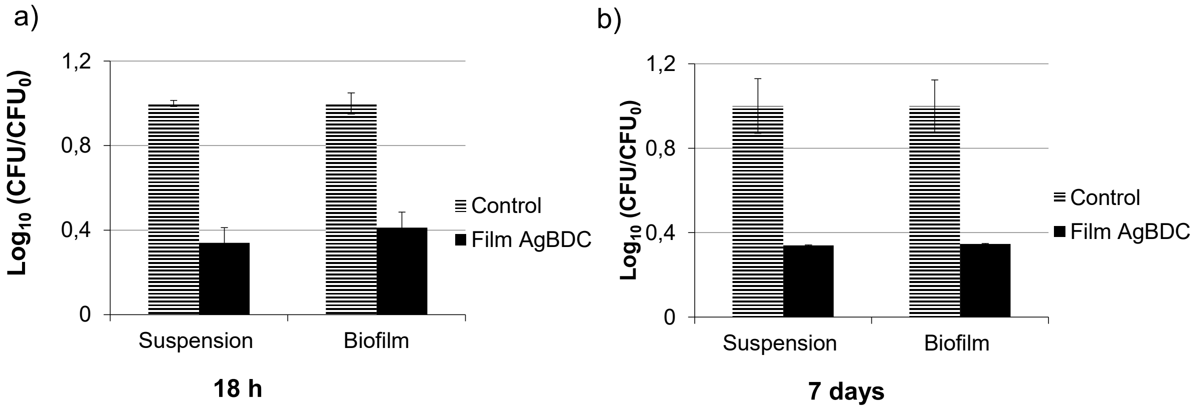

Figure S12. AgBDC thin film bactericide activity against E. coli after 18 h and 7 days of incubation at 37 °C (a) bacterial viability inhibition determined by OD_{600} and (b) enzymatic activity reduction determined from FDA fluorescent emission (λ_{ex}: 485 nm; λ_{em}: 538 nm).

Table S2. Compilation of Ag-based MOFs biocide activity against different microorganisims both in planktonic and sessile state.

MOF	MIC MOF ppm [Ag ppm]	Microoorganism	State	Reference
$[(AgL)NO_3]\cdot 2H_2O$<br>$[(AgL)CF_3SO_3]\cdot 2H_2O$<br>$[(AgL)ClO_4]\cdot 2H_2O$	300 [38] // 297 [38]<br>300 [35] // 307 [36]<br>308 [38] // 293 [37]	*E. coli* //<br>*S. aureus*	Planktonic	[25]
$Ag_3[C_7H_4O_5P]$	26 [16] // 26 [16] *1	*E. coli* //<br>*S. aureus*	Planktonic	[26]
$[Ag(\mu_3\text{-PTA=S})]n$<br>$(NO_3)_n\cdot nH_2O$	4 [1,2] // 20 [6] // 5 [1.5]	*E. coli* //<br>*S. aureus* //<br>*P. aureginosa*	Planktonic	[27]

$[Ag_4(\mu_4\text{-PTAL=S})(\mu_5\text{-PTA=S})(\mu_2\text{-}SO_4)_2(H_2O)_2]_n\cdot 2nH_2O$	20 [8] // 40 [16] // 20 [8]			
$[Ag_2(3\text{-NPTA})(bipy)_{0.5}(H_2O)]$	15 [6] //<br>20 [8]	*E. coli* //<br>*S. aureus*	Planktonic	[24]
$[Ag_2(O\text{-IPA})(H_2O)\cdot(H_3O)]$<br>$[Ag_5(PYDC)_2(OH)]$	10 [5] // 15 [7.5]<br>15 [9] // 20 [12]	*E. coli* //<br>*S. aureus*	Planktonic	[28]
AgTAZ	50 [30.5] //<br>50 [30.5] //<br>50 [30.5]	*Synechococcus*<br>*Anabaena*<br>*Chlomydomonas*	Planktonic	[29]
AgBTC	96 % //<br>90 % *2	*E. coli* //<br>*S. aureus*	Sessile	[14]
AgBTC	16 [2.4] //<br>32 [4.8]	*E. coli* //<br>*B. subtils*	Planktonic	[20]
AgBTC	50 [7.5] //<br>50 [7.5]	*E. coli* //<br>*B. subtils*	Planktonic	[30]
AgBTC	-	*E. coli*	Sessile	[15]
$AgBDC\text{-}NH_2$	-	*E. coli*	Sessile	[16]
Ag-2imi<br>Ag-Benzimi<br>Ag-imi	90 [13.5] // 85 [12.75]<br>75 [11.3] // 65 [9.8]<br>85 [12.8] // 80 [12]	*E. coli* //<br>*B. subtils*	Planktonic	[31]
$[Ag_2(O\text{-IPA})(H_2O)]\ (H_3O)$<br>$[Ag_2(bpe)_2(Cl)_2]$	5<br>25	*E. coli*	Planktonic	[21]
Ag-2imi	60% *2	*E. coli*	Sessile	[18]
$AgBDC\text{-}NH_2$	90% //<br>96% *3	*E. coli* //<br>*S. aureus*	Sessile	[17]
$Ag_2[HBTC][imi]$	100 [50] // 150 [75] //<br>25 [12.5]	*E. coli* //<br>*S. aureus* //<br>*P. aureginosa*	Planktonic<br>& sessile	[19]

Ag_2Cedcp<br><br>Ag_4Cmdcp	38 [18] // 38 [18] // 38 [18] // 38 [18]<br>10 [4] // 10 [4] // 10 [4] // 10 [4]	*E. coli* // *S. aureus* // *P. aureginosa* // *M. albican*	Planktonic	[32]
Ag-2imi	42 %<br>76 %*3	*E. coli*	Sessile	[33]
Ag-2imi	80 %<br>90%*3	*E. coli*	Sessile	[22]
Ag_6MTB_6	-	*E. coli* // *S. aureus*	Planktonic	[34]
$Ag_5(PYDC)_2(OH)$	-	*E. coli* // *S. aureus*	Planktonic	[23]
Ag-2imi	-	-	Sessile	[35]

*1 MBC; *2 % of reduction of CFU; *3 % of red cell on CLSM.

Legend: L: tris-(4-pyridylduryl)borane; PTA=S: 1,3,5-triaza-7-phosphaadamantane-7-sulfide; bipy : 4,4′-bipyridyl; H2NPTA : 3-/4-nitrophthalic acid; HO-H2IPA : 5-hydroxyisophthalic acid; H2PYDC : pyridine-3, 5-dicarboxylic acid; TAZ: triazole; BTC: 1,3,5-benzenetricarboxylic acid; BDC: Benzene 1,4-dicarboxylic acid; 2imi: methylimidazolate; H3CedcpBr :N-(carboxyethyl)-(3,5-dicarboxyl)-pyridinium bromide; H3CmdcpBr: N-(carboxymethyl)-(3,5-dicarboxyl)-pyridinium bromide; HMBT: 2-mercaptobenzothiazole.
